# Supplementary material for: Safety and Effectiveness of Abatacept in a Prospective Cohort of Patients with Rheumatoid Arthritis–Associated Interstitial Lung Disease
Source: Biomedicines. 2022 Jun 22;10(7):1480. doi: 10.3390/biomedicines10071480 (PMC9313094; doi:10.3390/biomedicines10071480)
Supplement: Supplementary file 1 [file biomedicines-10-01480-s001.zip › biomedicines-1738855-supplementary.pdf]

Supplementary Table S1. Previous treatment in patients with RA-ILD receiving abatacept.

Variable	Sample=57
Synthetic DMARD	43 (75.4)
Methotrexate, n (%)	24 (42.1)
Leflunomide, n (%)	19 (33.3)
Sulfasalazine, n (%)	12 (21.1)
Hydroxychloroquine, n (%)	8 (14.0)
Biologic DMARD	21 (36.8)
Infliximab, n (%)	2 (3.5)
Etanercept, n (%)	4 (7.0)
Adalimumab, n (%)	5 (8.8)
Golimumab, n (%)	1 (1.8)
Tocilizumab, n (%)	5 (8.8)
Rituximab, n (%)	4 (7.0)
Immunosuppressants	5 (8.8)
Mycophenolate, n (%)	2 (3.5)
Azathioprine, n (%)	1 (1.8)
Cyclophosphamide, n (%)	2 (3.5)

Abbreviations. RA: rheumatoid arthritis; ILD: interstitial lung disease; DMARD: disease-modifying antirheumatic drug.

Supplementary Table S2. *Lung function at diagnosis of ILD and at initiation of abatacept in patients with RA-ILD.*

Variable	Diagnosis of ILD	Baseline	p-value
FVC predicted (%), mean (SD)	80.4 (13.6)	76.5 (18.2)	0.042
FEV_1 predicted (%), mean (SD)	87.1 (16.9)	85.9 (10.4)	0.268
DLCO-SB predicted (%), mean (SD)	68.4 (16.2)	62.5 (16.5)	0.032

Abbreviations. RA: rheumatoid arthritis; ILD: interstitial lung disease; SD: standard deviation; FVC: forced vital capacity; FEV_1: forced expiratory volume in the first second; DLCO: diffusing capacity of the lung for carbon monoxide.

Supplementary Table S3. *Pulmonary function and progression at diagnosis of ILD and during follow-up.*

Variable	Diagnosis of ILD	Baseline	12 months	End of follow-up
Pulmonary function testing				
FVC predicted (%), mean (SD)	80.4 (13.6)	76.5 (18.2)	76.3 (16.1)	75.7 (14.4)
FEV_1 predicted (%), mean (SD)	87.1 (16.9)	85.9 (10.4)	85.1 (19.1)	84.7 (13.9)
DLCO-SB predicted (%), mean (SD)	68.4 (16.2)	62.5 (16.5)	60.6 (16.0)	59.7 (15.7)
Progression on HRCT				

Progression, n (%)	-	20 (35.0)	10 (17.5)	13 (22.8)
Stabilization, n (%)	-	34 (59.6)	41 (71.9)	39 (68.4)
Improvement, n (%)	-	3 (5.3)	6 (10.5)	5 (8.8)
Progression of lung disease overall*				
Improvement, n (%)	-	3 (5.3)	8 (14.0)	6 (10.5)
Stabilization, n (%)	-	28 (48.4)	39 (68.4)	35 (61.4)
Worsening, n (%)	-	26 (45.6)	10 (17.5)	13 (22.8)
Death, n (%)	-	-	-	3 (5.3)

*Abbreviations. RA: rheumatoid arthritis; ILD: interstitial lung disease; SD: standard deviation; FVC: forced vital capacity; FEV$_1$: forced expiratory volume in the first second; DLCO: diffusing capacity of the lung for carbon monoxide. *Progression of lung disease overall: considering HRCT and pulmonary function testing (FVC and DLCO).*

Supplementary Table S4. Characteristics of patients with RA-ILD who discontinued abatacept.

Patient	Age (years)	Treatment	Time since diagnosis of ILD (months)	Time receiving abatacept (months)	Reason for discontinuation
Patient 1	69.6	Abatacept and methotrexate	83.7	63.2	Recurrent urinary infection and joint failure
Patient 2	52.8	Abatacept and sulfasalazine	103.8	35.6	Joint failure
Patient 3	59.4	Abatacept and Hydroxychloroquine	78.1	72.0	Joint failure
Patient 4	60.0	Abatacept and sulfasalazine	88.7	31.0	Joint failure
Patient 5	80.7	Abatacept and hydroxychloroquine	56.2	12.3	Death: Progression of ILD and pulmonary superinfection
Patient 6	50.3	Abatacept and mycophenolate	49.8	47.7	Joint failure
Patient 7	78.5	Abatacept and hydroxychloroquine	108.2	41.1	Death: Progression of ILD
Patient 8	79.5	Abatacept and leflunomide	44.3	14.5	Death: Progression of ILD

Abbreviations: RA: rheumatoid arthritis; ILD: interstitial lung disease.

Supplementary Table S5. Factors associated with progression of lung disease in patients with RA-ILD treated with abatacept.

Variable	Improvement/stabilization (n=41)	Progression/death (n=16)	p-value
Epidemiological characteristics			
Sex, female, n (%)	21 (51.2)	11 (68.8)	0.231
Age, years, mean (SD)	67.6 (14.9)	67.8 (11.6)	0.970
Caucasian race, n (%)	41 (100.0)	16 (100.0)	1.000
Clinical and laboratory characteristics			
Smoking			0.677
Never smoked, n (%)	28 (68.3)	10 (62.5)	
Smoked, n (%)	13 (31.7)	6 (37.5)	
Duration of RA, months, median (IQR)	98.3 (63.7-230.9)	139.0 (70.6-200.9)	0.750
Duration of ILD, months, median (IQR)	45.0 (17.7-93.1)	53.5 (39.3-95.8)	0.390
Positive RF (>10 U/ml), n (%)	40 (97.6)	14 (87.5)	0.187
ACPA (>20 U/ml), n (%)	36 (87.8)	12 (75.0)	0.234
Inflammatory activity			
DAS 28-ESR, mean (SD)	3.0 (1.2)	4.4 (1.6)	0.012
HAQ, median (IQR)	0.8 (0.2-1.6)	1.0 (1.0-1.3)	0.503
C-reactive protein (mg/dl), median (IQR)	6.7 (5.0-13.1)	20.0 (6.1-32.0)	0.015
ESR (mm/h), median (IQR)	24.5 (11.7-41.5)	36.0 (15.0-55.0)	0.350
Radiological pattern			0.435
UIP, n (%)	28 (68.3)	8 (50.0)	
NSIP, n (%)	10 (24.4)	6 (37.5)	
Fibrotic NSIP, n (%)	3 (7.3)	2 (12.5)	
Baseline PFT			
FVC predicted (%), mean (SD)	81.0 (12.9)	66.9 (23.9)	0.013
FEV_1 predicted (%), mean (SD)	84.0 (12.6)	75.1 (28.2)	0.145
DLCO-SB predicted (%), mean (SD)	72.7 (12.3)	57.0 (15.5)	0.040
Treatment			
Time to initiation of abatacept, median (IQR)	22.1 (12.0-41.2)	34.0 (17.5-52.4)	0.109
Time receiving abatacept, median (IQR)	32.0 (17.5-42.4)	24.6 (10.7-40.8)	0.103

Combined with a DMARD, n (%)	32 (78.0)	13 (81.3)	0.790
Methotrexate, n (%)	19 (46.3)	3 (18.8)	0.044
Leflunomide, n (%)	12 (30.0)	5 (31.3)	0.927
Sulfasalazine, n (%)	1 (2.6)	1 (6.3)	0.507
Hydroxychloroquine, n (%)	3 (7.3)	3 (18.3)	0.406
Combined with immunosuppressants, n (%)	4 (10.0)	1 (6.3)	0.081
Mycophenolate, n (%)	2 (5.2)	1 (6.3)	0.890
Azathioprine, n (%)	2 (5.2)	0 (0.0)	0.570
Corticosteroids, n (%)	25 (61.0)	14 (87.5)	0.041
Dose of corticosteroids (grams), median (IQR)	2.5 (0.0-5.0)	5.0 (5.0-10.0)	0.037

Abbreviations. RA: rheumatoid arthritis; ILD: interstitial lung disease; SD: standard deviation; IQR: interquartile range; RF: rheumatoid factor; ACPA: anticitrullinated peptide antibody; DAS28: 28-joint Disease Activity Score; ESR: erythrocyte sedimentation rate; HAQ: Health Assessment Questionnaire; UIP: usual interstitial pneumonia; NSIP: nonspecific interstitial pneumonia; PFT: pulmonary function testing; FVC: forced vital capacity; FEV_1*: forced expiratory volume in the first second; DLCO: diffusing capacity of the lung for carbon monoxide; SB: single breath; ABT: abatacept; DMARD: disease-modifying antirheumatic drug.*
